# The what, the when and the how: A qualitative study of allied health decision‐maker perspectives on factors influencing the development and implementation of advanced and extended scopes of practice in Australia

**DOI:** 10.1002/hpm.3850

**Published:** 2024-10-03

**Authors:** Sharon Downie, Belinda Gavaghan, Megan D’Atri, Liza‐Jane McBride, Andrea Kirk‐Brown, Terry P. Haines

**Affiliations:** ^1^ The Royal Children's Hospital Melbourne Victoria Australia; ^2^ Executive Health Management PhD Candidate Monash University Melbourne Victoria Australia; ^3^ Office of the Chief Allied Health Officer Clinical Excellence Queensland Queensland Government Brisbane Queensland Australia; ^4^ Department of Paramedicine Monash University Frankston Victoria Australia; ^5^ Office of the Chief Allied Health Officer Clinical Excellence Queensland Queensland Government Brisbane Queensland Australia; ^6^ Department of Management Monash Business School Monash University Clayton Victoria Australia; ^7^ Head of School School of Primary and Allied Health Care & National Centre for Healthy Ageing Monash University Frankston Victoria Australia

**Keywords:** advanced scope, allied health, extended scope, qualitative inquiry, scope of practice

## Abstract

**Background:**

Health workforce supply is critical to ensuring the delivery of essential healthcare and may be enhanced via mechanisms which alter the scopes of practice of health professions. The aim of this paper is to study the collective perspectives of allied health decision‐makers on factors which influence their development and implementation of advanced and extended scope of practice initiatives, and how they contribute to scope of practice change. The reasoning for the selection of each factor will also be examined.

**Methods:**

A grounded‐theory, qualitative study of the experiences of allied health directors and senior managers across two Australian State/Territory jurisdictions.

**Results:**

Twenty allied health decision‐makers participated in the study. Data coding of interview transcripts identified 14 factors specific to scope of practice change, spanning rational (*n* = 8) and non‐rational (*n* = 6) decision‐making approaches. *Leadership*, *Governance*, *Needs of* o*rganisational leaders*, *Resourcing*, *Knowledge, skills & experience ‐ clinical*, *Supporting resources*, *Knowledge & skills – change* and *Sustainability* were identified as being rational and enabling in and of themselves, with *Leadership* seen as being most influential. Comparatively, the non‐rational factors of *Socio‐economic & political environment*, *Perceived patient need*, *Organisational environment*, *Change culture & appetite, Perceived professional territorialism* and *Actual professional territorialism* were more varied, and primarily influenced the timing/catalyst and application of decision‐making. The complex interplay between these factors was conceptually represented as a decision‐making construct.

**Conclusion:**

Allied health decision‐makers hold a complex, systems‐level understanding of scope of practice change. Whilst rational decision criteria were predominant and seen to enable scope change, non‐rational influences reflected greater variation in decision timing/catalyst and application, thus emphasising the human dimensions of decision‐making. Further research is required to better understand how decision‐makers integrate and weight these decision‐making factors to determine their relative importance and to inform the development of structured decision tools.

## Highlights

- Allied health senior decision‐makers hold a complex, systems‐level understanding of scope of practice change.
- Both rational and non‐rational factors are evident in their decisions to pursue advanced and extended scopes of practice.
- Rational factors are generally enabling of scope change in and of themselves, as compared to non‐rational factors which are more varied and generally reflect the timing/catalyst and application of decision‐making.
- Further research is required to better understand how decision‐makers integrate and weight factors in their decision‐making to determine the relative importance of individual factors and inform the development of structured decision tools.

## 1 INTRODUCTION/BACKGROUND

Mechanisms which alter the scopes of practice of health professions are recognised as a viable approach to critical workforce shortages in healthcare and have gained increased traction post the COVID‐19 pandemic.^1^ Scope of practice refers to the totality of functions, responsibilities, activities and decision‐making capacity that a health professional is educated, competent and authorised to perform within their role, and is conceptualised as a skills continuum, from standard care tasks requiring a common minimum skill‐set, to advanced and extended practice competencies with tasks restricted prior to successful completion of training and/or credentialling.^2^ Defining and building a profession's scope is not innovative in and of itself, rather it is the potential to vertically‐transfer tasks within or between disciplines (via task substitution from a more to less costly professional group, or task delegation from a more to less qualified workforce^3^) that theoretically provides opportunities to improve service access, efficiency and productivity.^2^

The breadth and depth of the allied health workforce, and their high levels of professional training, knowledge and technical skill‐sets, particularly lends these professions to assuming various clinical tasks traditionally viewed as the domain of medicine or nursing. Allied health is a functional categorisation used to collectively describe a number of professional‐level workforces which provide assessment, diagnostic, therapeutic and/or clinical interventions focused on physical, sensory, psychological, cognitive and social dimensions of health.^3^ The term gained prominence in the 1990s and remains in common usage in Australia, New Zealand, the United Kingdom and select countries within the European Union and Asia, despite there being no universally accepted definition, nor agreement as to the inclusion and exclusion of specific professional groups.^4^ A core of established allied health professions (including physiotherapy, occupational therapy and podiatry) has been recognised since the early 20th century, initially as ‘paramedical’ professions subject to medical authority, and now as autonomous professions with limitations of practice specific to parts of the body and/or specialised techniques.^4^ These established professions and emerging allied health professions (such as rehabilitation counselling and sonography) continue to evolve their practice to meet changing societal needs and technologies.^4^

Task substitution and delegation approaches have been widely adopted across allied health professions to progress their evolution, including as part of Australia's COVID‐19 vaccination programme.^5^ Advanced and extended scope tasks enable allied health professionals to work at their ‘upper limit’, effectively sharing or assuming tasks for other professional‐level workforces.^6^, ^7^ Advanced scope refers to an increase consistent with current scope of the profession but historically the remit of other professions, whilst extended scope denotes an increase that exceeds the profession's traditional scope but is supported through regulatory or legislative change.^8^ Enabling health professions to iteratively evolve beyond their traditional scope is purported to improve service access and resource utilisation at a systems‐level.^6^

Significant organisational investment is required to introduce and sustain an expanded scope of practice,^7^ and specifically the development and implementation of governance models, credentialling standards, education/training resources and competency assessments, and parallel stakeholder engagement and change management. This invariably requires significant human resources across multiple stakeholders, including healthcare organisations, professional regulatory bodies, peak associations and industrial partners. Similarly, there is a need for senior allied health and organisational leaders to have the requisite skills and capabilities, motivation and vision to realise these care models.^7^ These resource requirements, in addition to the time impost of workforce upskilling, carry a significant opportunity cost (that is, the ‘*opportunity to use those resources for another purpose*’^9^) which is exponential when replicated across multiple tasks, workforce groups and organisations. It is not immediately clear how decisions are best made to pursue advanced and extended scopes of practice models, nor if the perceived benefits outweigh the direct/indirect costs implicit in their operationalisation.

The purpose of this study is to identify the collective perspectives of allied health directors and senior managers within Australian public health services, as an exemplar healthcare workforce, on factors which influence their development and implementation of advanced and extended scopes of practice. This includes the reasoning underpinning their selection of influencing factors and how these factors contribute to scope of practice change. Study results will improve understanding of key decision criteria currently used to alter the boundaries of allied health scopes of practice. Application of these findings to practice scope decisions is critical to leveraging maximal health system capacity within resourcing constraints.

## 2 METHODOLOGY

### 2.1 study design

This qualitative study utilises a grounded‐theory approach and focuses on the experience of allied health directors and senior managers in the context of advanced/extended scope of practice roles. This approach is aligned to a constructivist epistemological research position, to describe and clarify influencing factors on advanced/extended scope of practice from the perspective of senior allied health leaders.^10^

### 2.2 Participants and setting

Eligible participants were allied health directors and senior professional/operational managers (‘allied health decision‐makers’) employed within public health services across two Australian states (Queensland and Victoria), with decision‐making responsibility for advanced or extended scope of practice roles. Participants were drawn from leadership roles across metropolitan, regional and rural health services within each jurisdiction, to reflect differences in experiences of decision‐making and variation in organisational size, service profile and geography.

The Australian healthcare system is based on a hybrid public‐private service model, with the eight State/Territory governments holding responsibility for the delivery of hospital operations within their jurisdictions.^11^, ^12^ Victorian and Queensland public health services were selected on the basis that their respective governance models provide a degree of contrast and both jurisdictions have provided funding for allied health advanced/extended scope initiatives. The Victorian Department of Health operates within a devolved‐governance model with central government assuming the role of system steward, as compared to Queensland Health, which whilst still devolved in structure, has greater centralised influence over clinical operations, quality and innovation.^13^, ^14^ A practical example of the delineation between these two governance models may be drawn from falls prevention, with Queensland Health implementing centralised policy initiatives across all health services within its jurisdiction, as compared to Victoria where individual health services are authorised to determine strategy specific to their local context.^15^, ^16^, ^17^

Exclusion criteria were defined as participant current employment within private/non‐Government organisations and sectors other than health, and less than six months overall leadership experience.

### 2.3 Procedure

Ethics approval was obtained via the Monash University Human Research Ethics Committee. An invitation to participate in the study and research explanatory statement were broadly circulated to allied health professionals employed in public health services in each jurisdiction by the Victorian Department of Health and Queensland Health. Interested participants meeting eligibility criteria were then formally consented by one of the Chief or Associate Investigators. The research explanatory statement advised participants of the broad aims of the research.

**FIGURE 1:**
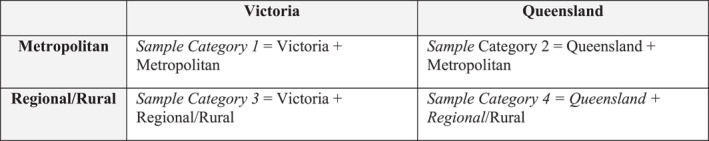
Sampling categories aligned to the use of bi‐dimensional maximum variation sampling.

Bi‐dimensional maximum variation sampling was used to source eligible participants, reflecting heterogeneity in organisational size, service profile and geography.^18^ A listing of all individuals who volunteered to participate was maintained by the Chief Investigator, noting their jurisdiction and geographic location to form four sampling categories. Figure 1.

Decision‐makers were interviewed in turn until a minimum of five participants had been sampled within each of the four sampling categories, or until such time as the populations within each category were exhausted. On this basis, two individuals who volunteered to participate in the study and otherwise met the inclusion criteria were not interviewed.

Pre‐determination of sample size was supported by research indicating that most qualitative datasets reach saturation between nine to 17 interviews (mean = 12–13).^19^

### 2.4 Instrumentation

Individual semi‐structured interviews were conducted with eligible participants by the Chief Investigator (Author 1) and Associate Investigators (Authors 2 and 3). All interviewers were female and had prior formal research experience. Authors 1 and 2 additionally held health leadership roles within their State/Territory jurisdictions (that is, Victoria and Queensland respectively) and also had lived experience of developing and implementing advanced/extended scope of practice models. Given their respective leadership roles, Authors 1 and 2 only consented individuals and conducted interviews outside of their home State/Territory and with individuals with whom they had no prior relationship.

An initial set of interview questions were developed, aligned to the stages of the Transtheoretical Model of Health Behaviour Change.^20^ Open‐ended questioning was primarily used to elicit information, with closed questions used to clarify responses. Questions were periodically reframed in response to new understandings gained from iterative data coding. Refer to Supporting Information for further details of the initial set of interview questions.

All interviews were conducted by video‐conference using the Microsoft Teams platform. Interviews were audio‐visually recorded and transcribed using embedded ‘voice‐to‐text’ functionality. Transcripts were then corrected against the recording by the Chief Investigator and saved as a Microsoft Word file, which was then reference‐checked with each participant prior to being finalised.

### 2.5 Data entry, management and analysis

Final interview transcripts, participant consent forms and audio‐visual recordings were stored securely within the Monash University Lab Archives system, a password‐protected storage portal. Data extraction was undertaken by the Chief Investigator using NVIVO qualitative data analysis software – version date 2020 (QSR International Pty Ltd., Melbourne Australia). Interview transcriptions were uploaded to NVIVO and reviewed as per the thematic analysis process outlined by Braun and Clarke.^21^, ^22^ Participant quotations were ascribed one or more codes to interpret their meaning. This process was replicated for each interview undertaken with data coded iteratively. The Chief Investigator continued to review and refine coding labels for the entirety of the data analysis process.

Codes were also used to identify the type of reasoning underpinning the participants' decision‐making, that is facts, logic and objectivity (so‐called rational decision factors)^23^, ^24^ versus other internally/externally generated factors (non‐rational decision factors).^25^

Themes were developed from final review and grouping of codes into like units of meaning, and were again analysed as to how they collectively contributed to scope of practice change ‐ that is factors which were viewed as enabling in and off themselves (the ‘what’ of change), influencing the timing or catalyst for change (the ‘when’) and/or influencing the performance of change (the ‘how’).

Member‐checking was undertaken to test the credibility of the coding process. Individual participants were provided with a sample of five to 10 codes from sections of their transcript. Participant feedback indicated agreement with coding samples provided.

The outcome of this thematic analysis is presented in detail within the Results section (see below).

## 3 RESULTS

### 3.1 Participant demographics

Twenty‐two allied health decision‐makers met the study eligibility criteria, with 20 subsequently participating in interview as per sampling category caps. The majority of participants were female (75%). Leadership experience of the cohort ranged from five to 40 years (mean: 16.9 years; median: 15.5 years), with a majority holding professional governance and/or operational responsibility for inpatient acute/subacute allied health services within large, networked health services. Only 4 (20%) individuals continued to practice clinically in their leadership role. Table 1.

**TABLE 1:** Participant demographics.

	Participant details
Gender	Male = 5 Female = 15
Role focus	Allied health leader = 11 Profession‐specific leader = 9
Professional background	Audiology = 1 Dietetics = 2 Occupational therapy = 2 Physiotherapy = 5 Podiatry = 2 Psychology = 2 Social work = 2 Speech pathology = 4
Leadership experience	Mean = 16.9 years Median = 15.5 years Range: 5–40 years
Continuation of direct clinical practice	Yes = 4 No = 16
Practice setting	Quaternary = 4 Tertiary = 6 Regional centre = 8 Rural/sub‐regional = 1 Networked metro/regional = 1
Organisational size	<100 inpatient beds = 1 100–500 inpatient beds = 3 500–1000 inpatient beds = 8 1000–1500 inpatient beds = 6 1500–2000 inpatient beds = 1 >2000 inpatient beds = 1
Geographic location	Metropolitan = 11 Regional/rural = 10 Note: participant (*n* = 1) aligned to a networked metro/regional provider
Jurisdiction	Queensland = 10 Victoria = 10

### 3.2 Thematic analysis

Inductive analysis of interview transcripts initially yielded a total of 41 codes related to decision‐making, with saturation achieved on the twelfth interview – this was arbitrarily defined as three consecutive interviews across categories with no addition of factors. The 41 codes were then reviewed and grouped by meaning to generate 14 themes reflecting key decision‐making factors. Refer to Supporting Information for a summary of theme development from data coding categories.

These 14 decision‐making themes or factors are described in order of frequency at Table 2 and discussed in detail below. Definitions of meaning constructed for each theme are provided at Table 2. A high‐level overview of the underlying reasoning underpinning each factor and its contribution to scope of practice change is also presented, along with a decision‐making construct that emerged via the grounded theory methodology.

**TABLE 2:** Descriptors of influencing factors on scope of practice decision‐making as identified by participants.

Decision‐making factor/theme	Definition of meaning	Predominant decision‐making approach		Why	
			What	When	How
			*Factor is an enabling influence in and off itself*	*Factor influences timing/catalyst*	*Factor influences application*
Leadership	Influence of the multi‐level leadership hierarchy within the organisation	Rational	×		×
Governance	Governance oversight of clinical practice both within the organisation and the broader environment	Rational	×		
Needs of organisational leaders	Organisational need as expressed by the leadership of the organisation	Rational	×	×	
Socio‐economic & political environment	Macro social, economic and political factors that create an environment for change	Non‐rational	×	×	
Perceived patient need	Needs of the patient or patient group as perceived by the allied health decision‐maker	Non‐rational	×		×
Resourcing	Human and financial resourcing to undertake activities related to scope of practice change	Rational	×		×
Knowledge, skills & experience ‐ clinical	Clinical knowledge, skills and experience that are required for the change in scope of practice to be performed	Rational	×		×
Supporting evidence & resources	Supporting evidence and resources specific to the proposed change in practice	Rational	×		×
Knowledge & skills ‐ change	Knowledge and skills that are required to operationalise and manage the scope of practice change process	Rational	×	×	
Sustainability	Sufficient human and financial resourcing and support to maintain the scope of practice change in the longer term	Rational	×	×	×
Organisational meso‐environment	Meso factors within the organisational context that facilitate change	Non‐rational		×	×
Change culture & appetite	Attitudes and opinions expressed towards changing practice	Non‐rational			×
Perceived professional territorialism	Perceptions that another health profession owns specific activities and would be adverse to sharing those activities with other professions	Non‐rational		×	×
Actual professional territorialism	Health professionals actively expressing views that would limit other professions performing activities that they would otherwise perform	Non‐rational		×	×

*Note*: Definitions.

Meso – influence on decision‐making occurring within institutional structures (that is, the health service)^26^.

Macro – influences on decision‐making operating at a societal level^26^.

#### 3.2.1 Leadership

Decision‐makers viewed leadership as being the strongest influence on scope of practice decisions and described this as consisting of two ‘nested’ elements – general leadership capabilities, and skills specific to stakeholder engagement and management.

The analysis identified solid leadership skills as being fundamental to enacting scope change within a single profession or collectively across professions. Participants viewed their leadership as encompassing a number of diverse elements, including environmental scanning of contemporary practice, exploration of funding sources, preparation of business cases and governance approvals, and change management. Consolidation of skills gained through professional experience and recurrent exposure to decision‐making contexts was viewed as beneficial in supporting successive efforts to advance or extend practice:

*‘I think the leadership point is really important. You need to have some senior leadership there* [participant when referring to themself]*, to give confidence and to give permission … and to negotiate the sticky situations when they come up. You leave that to a more junior group of individuals with less authority, it’s probably not going to happen’* (P7).

Decision‐makers also identified micro‐skills related to stakeholder engagement and management as being a critical leadership element. Participants spoke of the importance of simultaneously engaging stakeholders at multiple levels – above, below and across operational reporting‐lines. They consistently highlighted the benefits of leveraging existing relationships and interconnections, within their organisation and via professional networks, to mobilise support from internal leadership hierarchies and ensure the appropriate scale of proposed initiatives. Connection with stakeholders with decision‐authority was noted to be enhanced through visibility of leadership, sustained engagement, coalition‐building and strategic focus:

*‘I think that the challenge is … how you engage with stakeholders* [to negotiate changes to scope of practice boundaries] *… you might be crossing into their lane a little bit … it's got be well thought out, well‐reasoned to ensure that all stakeholders are comfortable with where they land’* (P6).

Engagement with ‘on‐the‐ground’ clinical leaders was also viewed by decision‐makers as essential to ensuring ownership and buy‐in for any proposed change in individual or professional scope.

#### 3.2.2 Governance

Allied Health decision‐makers cited governance requirements for scope of practice change as occurring at legislative and regulatory levels, as well as internal to their organisations.

Legislative and regulatory conditions were framed as higher‐order governance requirements, and the first consideration when exploring if a specific clinical task could potentially be undertaken by an allied health workforce:

‘*I'm probably a bit black and white … in that unless there is a regulatory boundary that keeps it* [a clinical task] *out of your scope, then your scope is what you'*re *trained and able to do safely. And so that's a very big net … look at the regulatory boundaries and seek guidance, and if you don't get a knock‐back from the professional body or legislation or the insurer, for example, then it's in scope’* (P7).

In progressing contemporaneous expanded scope roles and models of care, the assistance of government health departments was seen as essential to navigate identified legislative and regulatory challenges and drive practice change.

Considerable variation was evident in the type and levels of governance described across decision‐makers and organisations. Decision‐makers working within large tertiary/quaternary services described more stringent governance requirements with ultimate decision‐authority often sitting external to allied health leadership:

‘*The decision‐making* [related to allied health scope of practice] *is often not mine to make, which is a paradox really … the whole system is set‐up for the doctors to approve to let go, not the other side, which is allied health endorsing to take responsibility*’ (P20).

In these settings, governance was often viewed as straddling a ‘fine line’ between appropriate risk management and administrative ‘red‐tape’. This was perhaps not a surprising finding given these organisations invariably have multiple layers of upper/middle management across numerous professional groups. In contrast, regional hospitals were often described as having a less bureaucratic approach to governance so as to expedite access to essential clinical services:

‘*I think one of the benefits of working regionally is that the sheer number of egos that you have to get through to affect change is significantly less*’ (P3).

Several decision‐makers also spoke to conceptual considerations of where a task sits on the scope of practice continuum at different time‐points, and how a task may ‘organically’ evolve from advanced/extended practice to within scope as it becomes standard for a majority of the profession. Specific to governance, one participant explicitly described the concept of ‘reverse decision‐making’ for scope change:

‘*When something's become ‘in scope’ practice and it's previously been credentialled* [and now is seen as standard practice and therefore not requiring credentialling] *… that's highly challenging … I've gone to our credentialling committee and said “we'*re *taking this out”, and it's like, well, “why would you do* that”?’ (P19).

#### 3.2.3 Needs of organisational leaders

Needs of organisational leaders were at the forefront of decisions to pursue scope of practice change. Decision‐makers frequently cited demand management, service access and wait‐list reduction as the ‘hooks’ on which to build an advanced/extended scope strategy:

*‘Our hospital is going through a lot of care reform … How do we do things differently beyond the hospital walls? How do we recover post COVID? … So alternative care pathways are seriously being considered now. And I feel like allied health, that’s our gameplay, that's where we can position ourselves'* (P1).

All participants demonstrated to varying extents an understanding of the business imperatives facing their respective organisations, with service efficiencies, reduction in duplication and optimisation of medical and nursing capacity reported as being influential drivers of change:

‘*We are going to have to do more and more of it* [advanced/extended scopes of practice] *because the medical workforce is not there … They'*re *going to give‐up some of their stuff* [scope of practice]*, but we* [allied health] *need to be clever … it needs to solve a problem*’ (P19).

The strategic alignment and benefit of expanded scope models were generally presented in terms of anecdotal case‐reports or process measures, with very few participants citing the need for more formal cost/outcome assessment of alternate models. This was also reflected in participant's limited use of economic concepts and terminology when describing organisational need and benefit.

#### 3.2.4 Socio‐economic & political environment

Participants identified the influence of various macro‐environmental conditions on their experiences of scope change. The importance of political context was strongly acknowledged by Queensland‐based decision‐makers, in particular the alignment between government policy directives and authorising environments for change:

‘*Understanding the political environment is one of the most important things … I remember sitting in front of the AMA* [Australian Medical Association] *… and they'*re *firing shots at you like nobody's business, picking holes* [regarding a proposed advanced scope for physiotherapy] *… Then when you have the Minister or the political arm going “we’*re *going to continue”* [supporting the proposed change]*, then that’s not a problem anymore*’ (P7).

Interestingly, political environment was not a pronounced factor for Victorian decision‐makers.

Queensland decision‐makers also emphasised the importance of geography. They spoke of the relationship between the risk of no or limited access to care, particularly in rural/remote communities, and the apparent willingness of health providers in these communities to lessen bureaucratic requirements seen to delay or restrict efforts to enhance scope:

‘*I used to work in remote locations … if I wasn't … pushing the* [scope of practice] *boundaries of what I can and can't do, then you wouldn't get an assessment and that kid wouldn't be seen for another six months*’ (P6).

Crisis as a catalyst for change was strongly identified by Victorian decision‐makers, who experienced prolonged community lockdowns and mass health workforce mobilisation as part of initial COVID‐19 containment efforts. Several participants described changes in scope introduced specifically in the context of the pandemic response, and how change was actively enabled through organisational shifts in mindset and risk tolerance:

‘*They* [health services] *were initially really tight‐reined around who did nasal swabs and testing* [during the COVID‐19 pandemic response] *… it was nursing and then a few allied health … And then once we were a little bit through the pandemic, it was like, does it matter?’* (P1).

#### 3.2.5 Perceived patient need

Perceived patient need was a strong enabling factor common to decision‐makers across both jurisdictions. Decision‐makers used the terms ‘need’ and ‘outcomes’ to describe any change perceived as being beneficial:

‘*The first time that I considered it* [to progress an advanced scope model]*, it would be really to improve the patient journey and outcomes for the patient’* (P3); and

*‘It's all about the patients’ need, and what are we doing to enable that for the patients* [in relation to scope of practice change]*’* (P10).

Patient need spanned various dimensions of care including access, timeliness, quality, safety, user‐acceptance and effectiveness:

*‘Between the time that you* [podiatry] *were seeing them* [the patient] *and recommending that they needed antibiotics, and before they actually could get to the GP … they were being admitted to hospital with deterioration’* (P8); and

*‘I know that the majority of my dieticians will stand next to a junior doctor … to make clinical decisions or make changes to something that we’ve implemented* [but requires medical endorsement] … *we have that skill* … *so why can’*t *we* [do it]’ (P15).

Decision‐makers appeared to draw on their own values when ascribing the benefits of expanded scope initiatives:

*‘That's how I try and treat the patients that I work with* [and consider my practice scope]*, as though it is a family member … and I think if you've always got that at your centre, you'*re *going to put the patient first and do what's right*’ (P6).

Only two decision‐makers referenced managing patient expectations regarding changes in service delivery associated with expanded scope models:

‘*I think the thing that’s really important, particularly in developing those* [advanced/extended scope] *models of care, is to appreciate that people are turning up to the ED expecting to see a doctor …*’ (P11); and

‘*They'*re *not happy with it when you say I'm going to divert you off to see a physio or a dietitian … but once they experience it* [receiving care from allied health]*, they'*re *pretty happy*’ (P19).

#### 3.2.6 Resourcing

Financial resourcing was universally acknowledged as facilitating new advanced/extended scopes of practice, and ultimately their longer‐term success. Access to financial support was described as providing for dedicated staffing to develop and implement models of care, governance resources, training packages and change management approaches required to leverage scope changes:

‘*Everyone gives their in‐kind time, but without … half to three quarters of a full person … probably for 12 months ‐ you’*re *probably not going to get there* [realise an advanced/extended scope model]’ (P7).

One Victorian decision‐maker also identified budgetary implications aligned to state‐level industrial agreements for allied health professional employment:

*‘In our public agreement, you need to be* [paid] *at a certain grade level if you'*re *doing advanced scope’* (P17).

Decision‐makers also conversely spoke of the challenges when sufficient resourcing was not available, and the impact for realising and sustaining practice change:

‘*These things don't just happen* [new scope of practice models]*, so you do need to go out there and advocate strongly for funds … I’ve often sacrificed something in order to put a project officer in place for half a week for six months*' (P7); and

‘*We didn't have any project resource* [to support a new scope of practice model]*, and that was certainly one of the recommendations for next time … because it took too much time*’ (P18).

In this context, one decision‐maker voiced the opinion that allied health need to stop using funding as a barrier, and better utilise the resourcing that they do have in pursuing scope change:

‘*We absolutely need to start thinking about doing new things* [advanced/extended scopes of practice]*, incorporating new things in existing ways of working, not thinking of doing a new thing as a new stream of work. It's not*’ (P20).

#### 3.2.7 Knowledge, skills and experience‐ clinical

Clinical knowledge was consistently cited by decision‐makers as a core requirement for successful scope change. Decision‐makers described drawing on their own clinical expertise to inform their decisions, and how their decision‐making was often more certain for initiatives aligned to their own professional background:

‘*I think it comes back to your own clinical skills … what you have more familiarity with … Prescribing medications ‐ that for me, my approach* [to a new advanced/extended scope of practice] *would probably be a bit more rigorous. I would go line by line by line, “dot my i’s and cross my t’s” because it's not a comfort zone’* (P1).

In the context of clinicians assuming an expanded scope, knowledge and experience were viewed as two separate but inter‐related concepts. Clinical experience was viewed variously as being independent of hierarchical levels of knowledge and skill, with the implication being that expanded practice could be undertaken by all workforce levels commensurate with each clinician's knowledge base at a given time, as well as strictly tied to permissions to progress an advanced/extended scope:

‘*It's really this understanding that you can prescribe or do a particular skill as long as it’s within scope at the level commensurate with your level of understanding and skill at that time … It is not that you have to have a certain level of experience* [to perform advanced/extended scope tasks] *… as you gradually build your skills, all of these tools can be introduced*’ (P7); and

‘*They need to meet an absolute baseline of experience* [to assume an advanced/extended scope task] *… working or having achieved post‐graduate* [qualifications] *and working under the supervision of* … *senior leaders*’ (P12).

The temporal transition of clinical tasks within a profession, from novel to mainstream practice, was also seen to impact upon how the knowledge, skill and training needs of clinicians were conceptualised, and subsequent resource allocation decisions:

‘*I don't have the answer to this but … when does advanced practice no longer become “advanced practice”?’* (P1); and

‘*In a year's time it may not be advanced* [a specific clinical task]*, it might actually be standard scope of practice*’ (P13).

#### 3.2.8 Supporting evidence & resources

Decision‐makers made a clear distinction between internal and external sources of supporting evidence for scope of practice initiatives. Internal organisational data, such as patient quality/safety indicators and productivity measures, were noted as often providing the initial ‘problem definition’ and therefore impetus to pursue alternate care models and practice scopes:

‘[You] *look at things like incidents or impacts … of services not being provided, or limited services being provided … whether it be patient flow, length of stay, readmissions to hospital*’ (P9).

External benchmarking with other healthcare services, research and grey literature, programme evaluations, professional position statements and legislative documents were also seen as useful to better understand the potential application and likely effectiveness of practice scope change:

‘*Looking at the evidence‐base as well, and the literature, grey literature. Check in with our professional association to say ‐ “Is this within scope?” “Is it advanced versus extended?” “Is there legislative requirements here?”*’ (P1).

Decision‐makers however acknowledged that such external evidence was rarely aligned to their specific organisational setting and invariably needed to be contextualised to their local practice environment:

‘*Trying to find the most current up‐to‐date evidence* [of how a profession’s scope of practice is evolving] *… there's nothing in some areas … Then trying to correlate something that's kind of similar in another profession … It's hard to convince that, you know, that's going to work in our environment’* (P13).

In pursuing successive scope of practice changes, several decision‐makers also spoke to how learnings from past initiatives, and the formation of communities of practice, then informed future attempts often with significant time‐savings:

‘*I've developed now sort of a process to work through* [to address governance for scope of practice change] *… literature, benchmarking, you know, how is it being used elsewhere … so that defines our credentialling considerations*’ (P17).

This replication and sequencing of learning was thus seen as another evidence source (akin to a personal ‘toolkit’) which decision‐makers may actively draw upon, as distinct from understanding of change management more broadly.

#### 3.2.9 Knowledge & skills – Change

Distinct from clinical expertise, decision‐makers viewed knowledge of change management theory and methodologies as foundational to their own ability to successfully navigate change:

‘*I'm going to sound like a broken record, but the more I've seen people attempt expanded scope of practice, the more it's made me appreciate the importance of building a solid foundation to start with*’ (P5).

This was often expressed in terms of procedural knowledge or steps to be followed:

‘*I'm a pretty structured person, so I just follow the same way and start with establishing the need* [for the scope of practice change] *… Get your argument right, put safety things in place, … collect the data to make sure your outcomes are going to be effective … You need structures in place, and to get all these funds ready to go … And you turn up and you’*re *at the meetings, you’*re *presenting and you’*re *building networks*' (P7).

Similarly, decision‐makers also described the importance of building the change mindset and skills of ‘on‐the‐ground’ clinicians to realise, sustain and progress practice change:

‘*If they'*re *really onboard* [the clinical staff who will enact the scope of practice change]*, then you won't have to go through any significant change management process … if you've already got their buy‐in then it becomes more about logistics*’ (P9).

#### 3.2.10 Sustainability

Decision‐makers placed considerable emphasis on the sustainability of expanded scope initiatives, particularly in the context of the significant resourcing required;

‘*I always say to people, “what is your sustainability plan?” … There are models* [of care] *that stand‐up that rely on Sally to turn‐up to work in her yellow T‐shirt on a Tuesday to deliver that care,* [and] *when she doesn't turn up, we can't do it*’ (P19).

Two key factors were identified as impacting sustainability, reflecting both demand and supply‐side drivers. Sufficient patient demand, most usually related to the appropriateness of the model rather than a true lack of patients, was seen as being pivotal to longer‐term success, to not only justify resource allocation but also ensure maintenance of clinician skills:

‘*If it's going to be really person‐dependent and it's a minimal number of patients that would benefit from it* [the proposed scope of practice]*, we won't go forward*’ (P17).

Workforce pipelines were also identified as a key risk for service sustainability:

‘*If one person holds all that corporate knowledge* [underpinning a new scope of practice model] *… then as soon as they walk out the door it’s not going to be sustainable’* (P11).

This aligned with the significant focus that decision‐makers placed on staff training, namely mechanisms to acquire, retain and apply required knowledge and skills as part of an advanced/extended scope.

#### 3.2.11 Organisational meso‐environment

Decision‐makers identified a number of factors within their respective settings that supported the development and uptake of enhanced scopes of practice. Organisational size and structure were noted as being particularly influential. Working in a smaller organisation, with seemingly less complex governance requirements, was viewed by decision‐makers as facilitatory owing to the ability to better leverage interpersonal relationships and informal decision‐making to build a coalition for change:

*‘I can walk down this corridor and knock on the Director of Medical Services’ door. He knows who I am. I could have that conversation* [regarding a proposed scope of practice change]*. That would be a little bit more challenging in a larger metropolitan*’ (P3).

However, one participant from a sub‐regional facility also noted that less stringent governance may conversely lead to clinical risk:

‘*Rural health is a bit like people become “experts” because they'*re *the only person that can* [owing to availability] *… and that really sits very uneasy*’ (P14).

Two decision‐makers also noted the potential efficiencies to be gained if decisions were leveraged at a systems‐level, noting that many smaller hospitals have limited dedicated resourcing for clinical governance. In contrast, leaders in larger metropolitan organisations described how having a full complement of health professions, and comparatively less critical workforce shortages, shaped their decision‐making:

‘*We are very lucky in that there's often … someone else that can do it and do it quicker* [a clinical task that could be performed at advanced/extended scope by allied health] *… so, just because we could get approval to order imaging, order bloods, do something that's different …we don't need to*’ (P19).

As distinct from organisational culture, which is encompassed by the theme *Change culture and appetite*, several decision‐makers also spoke to previous organisational experience with expanded scope initiatives and how this then influenced subsequent attempts. Familiarity and success with similar models was described as creating a global‐sense of confidence and safety, thus providing for strong support and buy‐in across all organisational levels:

‘*I think a big factor there is probably confidence* [to adopt a new scope of practice model] *– and confidence from the organisation as well, that this will be supported and that we'*re *behind the person doing it*’ (P9).

#### 3.2.12 Change culture & appetite

The impact of culture was strongly evident in the scope of practice change examples provided by decision‐makers, and seemingly influenced change appetite at multiple and concurrent levels:

‘*The culture of the health service or health system that you'*re *working in has a big impact* [on interest in exploring scope of practice change]’ (P5).

Several allied health decision‐makers reported as having trained or worked internationally where scope progression was seen as being somewhat routine. Comparatively, they noted Australian healthcare organisations to be more conservative and less receptive to change:

‘*The ability to work to my previous full* [extended] *scope, having had that as an option in England … then having it as a bit of a barrier* [in Australia] *… it made me want to overcome it here*’ (P8).

Profession‐level culture, as well as the collective culture of allied heath, was also seen to influence scope change. Decision‐makers viewed the dominant culture of allied health as being largely risk averse:

‘*We've* [allied health] *been so downtrodden … we always ask permission rather than go and say “this is what we'*re *doing”* [regarding advanced/extended scope models]’ (P19).

Individual professions were perceived to vary in their acceptance of risk, from speech pathology who were described as being overly conservative, to physiotherapy who were viewed as being more action‐orientated:

‘*I've found that speech pathology as a profession is quite risk adverse*’ (P9); and

*‘I think of all those physios at the time* [pursuing an advanced scope of practice model] *… It was a coalition of the willing that really came together*’ (P11).

Patient harms and inferior quality care were key concerns for professions pursuing advanced and extended scopes of practice and reflected in the complex governance arrangements described. This point was expanded by three decision‐makers who attributed this internal bureaucracy to limited understanding of advanced/extended scope amongst allied health leaders themselves:

‘*I think* [allied health] *managers over time have lost the understanding* [of scope of practice] *… they don’t understand advanced prac*’ (P11).

As a result, governance mechanisms for endorsement were reported to often be the default to determine scope of practice level rather than to progress scope:

‘*I do see a lot of credentialling being done* [for allied health professions] *… for things which are really already in scope of practice … Credentialling committee for me is really around deciding something is definitely out of scope, recognised out of scope by the profession, and then giving that permission based on some governance and processes*’ (P7).

#### 3.2.13 Perceived professional territorialism

Perceived territorialism was identified as an implicit tension in altering scope of practice boundaries between professions, irrespective of whether this then manifested as actual resistance or conflict. Decision‐makers noted the potential for territorialism within and between allied health and other professional groups, and the concept of needing to ‘guard’ their core scope:

‘*Who's the gatekeeper?* [for scope of practice change at a profession level] *… Who's willing to give up some of their “stake in the land”? … If you'*re [an allied health profession] *not invested in the space and you leave it for too long, someone else* [another profession] *is going to come into that space*’ (P6).

Interestingly, implied territorialism was more evident in decision‐maker accounts of practice models that required skill‐sharing between allied health professions. Perceived loss of ownership for specific tasks or skills, and the perception of lesser standards of performance by another profession appeared to be the main drivers behind this dynamic.

#### 3.2.14 Actual professional territorialism

Five decision‐makers, across both jurisdictions, provided separate accounts of actual professional territorialism related to scope change, with all examples involving medicine and allied health.

The first decision‐maker spoke to resistance between medicine and dietetics at a systems‐level. They cited a lack of clarity as to the ‘hard boundaries’ for dietetics scope as contributing to limited support from medicine to relinquish discrete pathology ordering and prescribing tasks:

‘*The resistance* [towards allied health advanced/extended practice] *is by far from the medical field in just about every area that you want to look at. From a dietitian’s perspective, pathology requesting is actually considered in scope … There's limits, and I think no one seems to have worked out how to, how to circumvent them*’ (P3).

A second decision‐maker provided an account of medical territorialism as a barrier to the uptake of various expanded scope models across multiple organisations:

‘*Unless you get the doctors onboard* [with advanced/extended scope models] *and make it a systems approach, it will fall over. Because I've seen it time and time again, where we* [allied health] *stand something up and then the doctors don't like it, so they don't allow it to happen*’ (P19).

A third and fourth decision‐maker both noted resistance from medical trainees to the implementation of allied health‐led clinic models:

*‘I think they* [junior medical trainees] *were worried that they were losing some of their work* [to allied health advanced practice] *… feeling a little bit defensive’* (P13); and

‘*There was a bit of a worry at one point about “well, how are the Registrars* [junior medical trainees] *getting their* [training] *experience?”’* (P12).

Lastly, a fifth example from a decision‐maker in a large quaternary service described a long‐standing dispute between ear, nose and throat surgeons (ENT's) and speech pathologists regarding ownership for a specific instrumental test, fibreoptic endoscopic evaluation of swallowing (FEES). This conflict was reported to have played‐out for more than a decade and across changes in senior leadership for both professions. This is despite FEES, including passing of the nasendoscope, being acknowledged as ‘within scope’ by the professional association for Australian speech pathologists^27^ and routinely undertaken across other public hospitals:

‘*I've just got the battle of trying to get them* [the ENT’s] *to acknowledge that this* [FEES] *is in scope* [for speech pathologists]*. Every other health service organisation does*' (P1).

In exploring the reasons for this conflict, the decision‐maker cited a fundamental disagreement between the parties as to how FEES is conceptualised, with the ENTs of the opinion that ‘*passing a scope is a medical procedure*’. This is in direct contrast to the decision‐maker's perspective that ‘*if it was a medical procedure there would be a legislation requirement that it was only a doctor … it's a technician skill, it's a waste of their time – they are not interpreting the swallow, because that's not their role’.*

Refer to Supporting Information for supplementary quotes aligned to each theme.

### 3.3 Reasoning underpinning decision‐making factors

Eight of the 14 decision factors described by allied health decision‐makers were underpinned by facts, logic and objectivity and were therefore noted to be based in rationality.^23^, ^24^ These predominantly related to structural influences on decision‐making (for example, regulatory/legislative requirements and leadership delegations) or the presence/absence of physical resources (for example, funding, workforce, training and knowledge). The remaining six factors reflected internally/externally generated, non‐rational influences,^25^ namely decision‐maker preferences, beliefs, motivations and emotional responses, exemplified by descriptions of their own professional values, inter‐personal relationships, workplace cultures and power dynamics. Refer to Table 2.

### 3.4 Contribution of factors to scope of practice change

Analysis of each factor is relation to its contribution to scope of practice change revealed that a majority of factors enacted change through more than one mechanism, that is being enabling in and of themselves (‘what’), influencing the timing or catalyst for change (‘when’) and/or influencing the performance of change (‘how’). Only two factors (*Governance* and *Change culture and appetite*) were identified as being uni‐dimensional in their influence, respectively reflecting the ‘what’ and ‘how’ of change. *Sustainability* was the only factor seen to enact all three mechanisms of change. Refer to Table 2.

### 3.5 Integration of factors within a decision‐making construct

**FIGURE 2:**
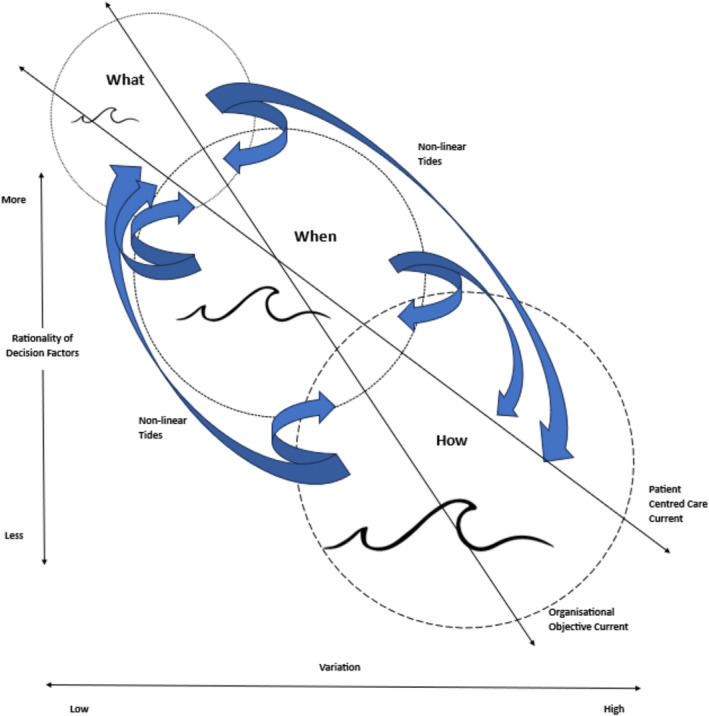
Conceptual representation of a scope of practice decision‐making construct.

Through the use of the grounded theory methodology, allied health decision‐makers’ descriptions of the 14 influencing factors, the rational/non‐rational criteria underpinning their selection, and the ‘what’, ‘when’ and ‘how’ of their influence were integrated within a decision‐making construct presented at Figure 2.

At a systems‐level, the interplay between the factors and their various dimensions of reasoning and influence can be conceptually represented as three concurrent and overlapping ‘waves’ of decision‐making – represented as three interconnected circles describing key iteration points in the decision‐making process, with the blue arrows used to convey the potential permutations of each decision and the black arrows highlighting common influencing variables. This conceptual model evolved from the way in which allied health decision‐makers explained the complexity of their decision‐making, that is the simultaneous but non‐linear interaction of multiple influencing factors within complicated operating environments – akin to a ‘churning ocean’.

The smaller, largely consistent wave of ‘what’ decision criteria (rational influences) gives rise to the progressively larger and non‐uniform swells of ‘when’ and ’how’ (non‐rational influences), which introduce greater variability in decision outcomes. ‘Tides’ operating within and between the decision‐making waves demonstrate the non‐linear nature of the decision process, and reflect descriptions provided by decision‐makers where factors are continually re‐evaluated through forward and backward ‘chaining’ to add and/or subtract factors to form a decision outcome. ‘Strong currents’ within the wider ‘decision‐making ocean’ illustrate how patient‐centred care (access, experience, clinical outcomes) and organisational objectives (efficiency, effectiveness) cut across all decision‐making factors and are common to decision‐maker perspectives.

A worked exemplar of this conceptual model using study data is available as Supporting Information.

## 4 DISCUSSION

This qualitative study of senior allied health decision‐makers has identified 14 decision factors specific to advanced and extended scope of practice and highlighted the complex nature of decisions relating to scope of practice change.

The factors of *Leadership*, *Governance*, Needs of o*rganisational leaders*, *Resourcing*, *Knowledge, skills and experience – clinical*, *Supporting resources*, *Knowledge & skills – change* and *Sustainability* predominantly reflected rational decision criteria, viewed as the ‘what’ of decision‐making, and enabling in and of themselves. Rational decision‐making is based on facts, logic and objective analysis and is often cited as the perspective by which effective decisions should be made.^23^, ^24^ For example, allied health decision‐makers cited new models of care encompassing scope change as a logical mechanism through which to manage the ‘what’ of organisational need, more specifically increasing demand for clinical services. However, several participants noted significant limitations in rational decision‐making approaches, namely the lack of local research evidence specific to scope changes under their consideration.

Although multiple studies suggest that expertise in a given field is correlated with more rational decision‐making approaches,^28^ the findings of this study of ‘expert’ allied health decision‐makers did not fully support this finding. *Socio‐economic & political environment, Perceived patient need, Organisational meso ‐environment*, *Change culture and appetite*, *Perceived professional territorialism and Actual professional territorialism* were more reflective of non‐rational approaches and generally described greater variation in the timing, or catalyst for change, (the ‘when’) and application (the ‘how’) of decision‐making. Non‐rational decision‐making encompasses the influence of internally and externally generated factors.^25^ Internal influences are specific to each individual decision‐maker and modulate how they cognitively‐process relevant information to form a decision outcome.^23^ An individual's emotional state (affect), previously‐held knowledge and experiences (heuristics), and perceived personal value of the potential outcome (personal utility) may all subjectively impact upon their decision‐making.^23^ This was evident in allied health decision‐makers’ descriptions of perceived patient need, and how their previous experiences and beliefs regarding the acuity or significance of that need appeared to inform when and how scope of practice change was operationalised.

The literature also cites a number of common external influences on non‐rational decision‐making, namely political climate, economic factors, legislative and regulative requirements, and stakeholder power.^29^ Pettigrew and Whipp describe external influences as the ‘why’ of organisational change, and further distinguish between inner and outer contextual influences.^30^ These factors were well‐represented in this study, with the ‘when’ and ‘how’ of decision‐making collectively describing the ‘why’ referenced by Pettigrew and Whipp. Inner context refers to meso‐level factors within an organisation, namely structure, size and culture, whilst outer context conversely describes macro‐level factors external to the organisation, such as the social, political and economic landscape.^25^, ^31^ The deliberate inclusion within this study of allied health leaders from different‐level hospitals and geographic locations in two Australian jurisdictions provided for comparison of these contextual factors, and demonstrated that smaller organisational structures (inner context) and regional locales (outer context) were indicative of an openness to pursue scope change. Health policy and regulatory and legislative approvals (outer context) were also viewed as significantly more influential than meso‐level organisational factors in gaining momentum for change.

The influence of non‐rational factors and evidence of non‐linear decision‐making (as described in Figure 2) both emphasise the human dimensions of scope of practice decision‐making. This challenges the tenets of Expected Utility Theory, which proposes that experienced decision‐makers are able to understand and rationally evaluate complex issues independent of other non‐rational biases and influences, to form preferred outcome judgements that maximise societal gain.^23^, ^24^, ^32^ This finding is supported by a number of authors who contend that failure to consider individual experiences, preferences and values in decision‐making impedes practice and policy implementation and the openness and transparency of these processes.^23^, ^28^, ^32^ Of the 14 factors identified, *Leadership* was the most prominent decision criteria influencing scope change, reflecting both the ‘what’ and ‘how’ of decision‐making. Leadership is widely acknowledged as being central to successful change management and the vehicle through which ‘strategising’ is translated to ‘organising’ to enact an organisation's identity, culture and interests.^33^, ^34^ Mid‐level leaders, such as allied health decision‐makers, are unique in their experience of change in that they are often concurrently recipients and drivers of change.^35^ Such leaders play an important role in supporting organisational change readiness, through first understanding organisational needs and directives from executive management, and then communicating and operationalising these directives through the actions and behaviour of their direct reports. Implicit in this dynamic is the understanding that change to a future desired state is not driven by organisations per se, but the people who encompass an organisation.^36^ Contemporary change management approaches therefore place significant emphasis on empowering individuals at all levels to navigate and respond to uncertain and unpredictable environments, with leadership strongly correlated with individual motivation and agency for change, and in turn organisational readiness.^37^ It is therefore not surprising that decision‐makers in this study placed significant value on the enabling qualities of leadership capability, both their own and that of other internal stakeholders.

### 4.1 Implications for practice

The results of this study provide a greater understanding of key factors which influence allied health advanced/extended scopes of practice, as well as the inherent complexity and variability of the decision‐making processes. Given that strategic, structural and resourcing decisions in healthcare environments are usually delegated to individuals who hold decision‐authority on behalf of a larger workgroup (such as allied health decision‐makers), it is important that organisations facilitate optimal decision‐making environments and provide their staff with decision skills training and structured tools which allow for consideration of both rational and non‐rational influences.^28^, ^38^ There is evidence that increased awareness of and attention to decision‐making process enhances decision outcomes and sustainable change^23^ and may mitigate individual and group‐level decision biases.^28^

### 4.2 Areas for future research

Further research is recommended to build on the findings of this study, specifically the underlying decision‐making processes utilised by allied health decision‐makers, and alignment to existing cognitive psychology and decision‐making models (such as Kahneman's System 1 and System 2 model^39^).

Early indications from this research suggest that allied health leaders are potentially influenced by the perceived ‘loss’ or ‘gain’ that they ascribe to various circumstances. For example, the lack of service access in remote communities was cited by several participants as a clear justification to proceed with various advanced/extended scopes and to offset other risks implicit in these practices. This builds on the principles of Prospect Theory, which describes the inherent ‘asymmetry’ of decisions involving a perceived ‘loss’ or ‘gain’, with the degree of the perceived loss/gain then influencing the proportionality of actions taken.^40^

Comparative review of the decision‐making processes of medical and nursing leaders would be of particularly interest to understand if allied health decision‐makers are unique in how they integrate and weight decision factors to determine the perceived costs/benefits of their decisions. Given that inappropriate weightings may result in disproportionate risk aversion/appetite,^28^ this research would assist the development of structured decision tools to support allied health decision‐makers, and potential other health workforce groups, to make optimal decisions which ultimately enhance healthcare service access.

### 4.3 Limitations

There are several limitations inherent in this study design which should be acknowledged. Grounded theory methodology, although advantageous in allowing for iterative thematic analysis to inform subsequent data collection and the emergence of meaning from the data, suffers from the disadvantage of non‐standardisation of process and so is not replicable. It is likely that the Chief Investigator's own lived experience of successfully leading large‐scale scope of practice change will have introduced a degree of subjectivity in their interpretation of some data, particularly in relation to identification of enabling factors. Lastly, the generation of a conceptual decision‐making model, whilst reflective of the study cohort's described experiences, may not reflect the experience of other allied health leaders and/or different practice contexts.

## 5 CONCLUSION

This qualitative study of senior allied health leaders highlights the inherent complexity in decisions to develop and implement advanced and extended scope of practice initiatives. Fourteen discrete factors were identified as explicitly influencing the ‘what’, ‘when’ and ‘how’ of scope of practice decisions at a systems‐level, with leadership being the most prominent enabling factor. Reliance on both rational and non‐rational decision‐making influences and non‐liner process amongst this group emphasises the importance of the human dimensions of decision‐making and challenges the assumption that subject matter experts are more likely to rely on rational decision approaches.

Further research is required to better understand how allied health decision‐makers integrate and weight various decision‐making influences, to inform the development and pilot of structured decision tools. Through providing decision‐making supports that clearly articulate and evaluate the relative costs/benefits of advanced/extended scope initiatives, it is envisaged that the rigour and consistency of decision‐making would be improved, ultimately resulting in optimal outcomes for healthcare consumers, organisations and society more broadly.

## CONFLICT OF INTEREST STATEMENT

The authors declare no competing interests.

## ETHICS STATEMENT

Not applicable.

## Supporting information

Supporting Information S1

Supporting Information S2

Supporting Information S3

Supporting Information S4

Supporting Information S5

## Data Availability

The data that support the findings of this study are available on request from the corresponding author. The data are not publicly available due to privacy or ethical restrictions.
