# Supplementary material for: The what, the when and the how: A qualitative study of allied health decision‐maker perspectives on factors influencing the development and implementation of advanced and extended scopes of practice in Australia
Source: Int J Health Plann Manage. 2024 Oct 3;40(1):130–55. doi: 10.1002/hpm.3850 (PMC11704828; doi:10.1002/hpm.3850)
Supplement: Supplementary file 1 — Supporting Information S1 [file HPM-40-130-s005.docx]

**Supporting Information:**

**Summary of theme development from data coding categories**

| **Codes** |  | **Themes** |
| --- | --- | --- |
| 1. Change management process 2. Crisis as catalyst 3. Leadership skills 4. Staff buy-in 5. Staff resistance to change 6. Stakeholder relationships 7. Health system understanding |  | *Leadership* |
| 1. Governance requirements 2. Decision authority 3. Legislation and regulation |  | *Governance* |
| 1. Resource efficiency 2. Hospital readmission 3. Patient length of stay 4. Patient flow and treatment delay 5. Service access and demand management |  | *Needs of organisational leaders* |
| 1. Political climate 2. Organisational funding 3. Rural, regional and remote locality 4. Societal need |  | *Socio-economic & political environment* |
| 1. Patient care values 2. Patient outcomes |  | *Perceived patient need* |
| 1. Training access 2. Staff resourcing 3. Training costs |  | *Resourcing* |
| 1. Clinical knowledge 2. Clinical skills |  | *Knowledge, skills & experience - clinical* |
| 1. Profession specific guidance 2. Research evidence and evaluation 3. Policies/procedures 4. Inter-organisational comparisons |  | *Supporting evidence & resources* |
| 1. Change management skills |  | *Knowledge & skills - change* |
| 1. Skill maintenance 2. Sustainability |  | *Sustainability* |
| 1. Within the hospital 2. Organisational culture |  | *Organisational meso-environment* |
| 1. Allied health cultures 2. International comparisons 3. Belief in own practice 4. Risk aversion |  | *Change culture & appetite* |
| 1. Perceived professional territorialism |  | *Perceived professional territorialism* |
| 1. Perceived professional territorialism |  | *Actual professional territorialism* |
