# Supplementary material for: The what, the when and the how: A qualitative study of allied health decision‐maker perspectives on factors influencing the development and implementation of advanced and extended scopes of practice in Australia
Source: Int J Health Plann Manage. 2024 Oct 3;40(1):130–55. doi: 10.1002/hpm.3850 (PMC11704828; doi:10.1002/hpm.3850)
Supplement: Supplementary file 2 — Supporting Information S2 [file HPM-40-130-s004.docx]

**Supplementary Information**

**Initial interview questions aligned to stages of the Transtheoretical Model of Health Behavior Change (Prochaska & Velicer, 1997)**

| **Stage of Change** | **Key questions** | **Potential prompts** |
| --- | --- | --- |
| Precontemplation to Contemplation | Within your current role (or a past role) have you identified a potential need for a scope of practice change for a professional group? Please outline the scope of practice change considered and practice context. | - for your own profession? - for another Allied Health profession? - for an Allied Health assistant workforce? - on a single/multiple occasions? |
|  | What issues / problems / situations influenced your thinking that a scope of practice change may be required? |  |
|  | Beyond your initial reasoning for the potential need for scope of practice change, did you consider other factors related to the proposed change?  If so, what were the factors that influenced you to consider the proposed change more seriously?  If not, what were the factors that influenced you not to proceed further with the proposed change? |  |
| Contemplation to Preparation | What factors influenced your decision to explore how to make the scope of practice change happen? |  |
| Preparation | In preparing to make the scope of practice change, what information did you seek to support your decision? | - research literature? - professional education? - discussion with sector experts? - consultation with internal/external stakeholders? - other? |
|  | Who/where did you seek this information from and why? | - individuals consulted? - organisations consulted? - order of consultation? - relationship to those consulted? |
|  | Did you refer to organisational procedures or policies to support your decision?  If so, did you apply factors listed within organisational procedures or policies to your decision-making process? | - why/why not? - respondent involvement in authoring or endorsing resources? - documents generic to all workforces or specific to Allied Health? - inclusion of all factors - why/why not? - exclusion of some factors - why/why not? |
| Preparation to Action | What factors influenced your decision to take action to make the scope of practice change happen? | - why are these factors significant? |
|  | Did you hold the ultimate authority for this decision?  If not, who was the decision-maker, and what was the process for providing them with information to authorise this decision? |  |
| Action | Did you lead the implementation of the scope of practice change or were other leaders also involved? | - other leaders involved? - professional roles? |
|  | What practical steps did you (or the collective leadership group) undertake to make the scope of practice change happen? |  |
|  | Where other individuals required to alter their behaviour to support the scope of practice change?  If so, what factors do you believe supported or obstructed behavioural change? | - other individuals involved? - personal factors? - organisational factors? - external environmental factors? |
| Maintenance | Was the change in scope of practice realised in practice? | - why/why not? |
|  | Has the change in scope of practice been maintained? | - in full/in part? - consistent/inconsistent application? |
|  | Has there been resistance to the change in scope of practice?   - If so, please outline the type of resistance encountered? How has this been managed? | - effectiveness of management strategies? |
| Other | Do you think that your understanding of decision-making factors has changed as a result of previously working through a scope of practice change process (either in part or in full)? | - do you now consider more or less factors in your decisions? - do you now apply different weightings to decision-making factors? |
|  | If participating in a future decision to change a scope of practice, would you now identify any additional factors? | - why are these factors significant? |
