# Supplementary material for: The what, the when and the how: A qualitative study of allied health decision‐maker perspectives on factors influencing the development and implementation of advanced and extended scopes of practice in Australia
Source: Int J Health Plann Manage. 2024 Oct 3;40(1):130–55. doi: 10.1002/hpm.3850 (PMC11704828; doi:10.1002/hpm.3850)
Supplement: Supplementary file 3 — Supporting Information S3 [file HPM-40-130-s003.docx]

**Supplementary Information**

**Supplementary quotes aligned to themes**

| **Theme/Sub-Theme** | **Supplementary Quotes** |
| --- | --- |
| Leadership | *‘I think it always comes down to knowing the motivation of our stakeholders* [when canvassing for scope of practice change] *and knowing the local context - what to push and what not to push’* (P1)  *‘I probably always knew that having champions, clinical champions was important, but I didn't realize how particularly important – particularly with changing scope’ (P7)*  *‘It can be a little person-dependent as to who is open-minded to these sorts of opportunities* [advanced/extended scope of practice] *… I had a consultant … who was just an absolute ally the whole way to enabling us to set-up shop there and prove what we could do and give us a chance’ (P11)*  *‘We had a key orthopaedic surgeon who was very proactive and supportive with the progressing of the model* [allied health advanced scope of practice]*. I think he had some challenges himself in his “own tribe”, if you like, trying to get everybody across the line, so I think there were a few … stops and starts from that perspective’ (P18)*  ‘*I always say to people* [in relation to role in scope of practice change]*, “I'm your red tape person, your barrier person, your connector” …*’ (P19)  ‘*The usual example is, you know, five Gastroenterologists, four onboard, and one not* [with allied health pursing advanced scope of practice tasks]*. Bring me in with that one, so that we can look at how we engage*’ (P19)  *‘I think it is about knowing your audience in terms of decision-making and who you’ve got to get online* [for allied health scope of practice change] *… there are different levels of, call it evidence or benefits statements, that you’ll use for different audiences’* (P20) |
| Governance | *‘It’s not a linear chain* [for approval of scope of practice change] *… Allied Health Scope of Practice, then led up to Allied Health Executive Committee - so there is linkage there for approval. It went to the Project Steering Committee ... It went to ICU Quality and Safety Committee as well. Then it went to Clinical Innovations Committee’ (P1)*  *‘The legislation* [understanding of] *– so that's where you need a lot of help from the Department to negotiate with various regulatory bodies to … provide an exemption or approval to the legislation to enable extended scope of practice … I've been to other regulatory bodies or representative bodies to explain the things that we’re doing* [extended scope of practice]*, and there’s not a lot of joy there. But at the end of the day, they won’t stop anything, can't stop anything. As long as the Minister for Health and the Department of Health are onboard* [with the extended scope of practice] *… those other interest groups just become interest groups and are an insignificant barrier’ (P7)*  *‘We definitely have a significant credentialing committee here* [for scope of practice] *… if you do want to introduce maybe an additional scope for a discipline, it's a very extensive process that we would have to go through’ (P9)*  *‘There was absolutely an expectation from those medical leads that we would have a very, very strong quality and compliance requirement* [for the introduction of allied health advanced/extended scope of practice]*’ (P12)*  *‘There was an appetite, and I think that was probably one of the* [most important] *things. There was a real appetite from the Department of Health for advanced practice’ (P11)*  *‘So many things* [clinical tasks] *that people talk of being advanced scope / extended scope isn’t … when I’m looking at examples of when it is extended scope, it’s something that you need to change the law to be able to do’ (P19)* |
| Needs of organisational leaders | *‘We’ve got a tired workforce* [post the COVID-19 pandemic response] *… nursing and medical saw it* [allied health advanced scope of practice] *as an opportunity for someone else to take some of their workload away, with little investment on their behalf’ (P1)*  *‘With our* [advanced scope] *physiotherapists in the emergency department, … 30 to 40 percent of patients they see have fractures or dislocations, so it’s just easier to stream them off … and extract the doctor for other things’ (P7)*  *‘It’s all about pitch I think* [for allied health advanced/extended scope models] *– in thinking about what they* [other stakeholders] *are ‘thinking’, and how does it impact them’ (P11)*  *‘We actually got our pilot* [of an advanced scope of practice] *extended because of an audit that we did on back pain that showed that if you saw a musculoskeletal physio you’re fifteen times less likely to be admitted … so that kind of got the attention of the Executive, who said “oh, this sounds good”. And we got the 7-day model based on that’ (P11)*  *‘The organisational factors, I think they're probably the stronger drivers now* [for approval of allied health advanced/extended scope models]*, and they're seen more widely as a stronger driver. What's the activity? How do we achieve our activity, our NWAU (National Weighted Activity Units)? So, really making sure that we've got the appropriate impact on patient flow, patient access, patient capacity and ensuring that we've got that those KPIs, those organisational-wide KPIs. How do we contribute to those? How do we help drive them? Perhaps earlier it was a bit more around we've got great people wanting to do great things’ (P12)*  *‘There was a recognition … that there are lots of category 3 and 4 presentations* [to the Emergency Department] *… These people* [patients] *coming in and presenting were a long way down the priority listings, so they were waiting for a long time and often being seen eventually by an intern* [junior doctor]*, who wasn't necessarily well-versed in managing these issues. So, that really came together in the perfect opportunity* [for allied health advanced scope of practice] *rather than the perfect storm’ (P12)*  *‘Staffing was a huge challenge* [to provide allied health services in a rural area] *… We actually didn't have the budget even … if we could find people* [allied health workforce] *… so, the use of an interdisciplinary* [allied health] *professional was much smarter’ (P14)*  *‘It is performance driven, not quality driven* [for approval of allied health advanced/extended scope models] *… it is only sparked from the organisation when there's a performance problem with waitlists or there's a reputational problem with media and negative media around the time it takes for people to get appointments. And then quickly, everybody you know, there's this flurry’ (P20)* |
| Socio-economic & political environment | *‘Reflecting on my Australian experience* [with dietetics-led pathology ordering] *… the Allied Health Professions Office of Queensland, this* [dietetics advanced/extended scope of practice] *is something that's been on their radar’ (P3)*  *‘In Queensland, probably going back 5, 6, 7 years, there was a big push* [on advanced/extended scope of practice roles] *… and the government had a political commitment to reduce waiting lists, and that provided a little bit more funding to introduce these roles’ (P7)*  *‘Darwin for instance - there probably was I think more ability to move things* [scope of practice change] *a bit faster in that space, because I think you just needed to. Otherwise, there were really going to be impacts for, you know, the type and the effectiveness of the services’ (P9)*  *‘We did get the Department of Health’s support to do some project work around the operational framework and the clinical education framework* [for implementation of an advanced scope of practice model]*’ (P11)*  *‘At that particular time, the Department of Health in Victoria was providing some funding to really explore, grow, develop the advanced practice role, so there are lots of drivers’ (P12)*  *‘We were fortunate enough … to get one of the Department of Health advanced practice grants. And the team at the time developed an advanced developmental paediatric role which has been hugely successful’ (P16)*  *‘There was a lack of barium availability during COVID which has continued. So, we were able to put forward to get some additional equipment for swallow assessment which could then be used for the laryngeal evaluation* [at an advanced practice level]*’ (P17)* |
| Perceived patient need | *‘With that particular client group, they're already coming weekly, or sometimes twice weekly. That's a huge commitment. To add further trials and tribulations, to go off and access your GP or go somewhere else* [when an advanced scope role could provide all care]*, would just add to their burden’ (P3)*  *I've actually come away from the “we're clever, look how clever we are, we can do this* [assume clinical tasks historically completed by medicine]*”, to “what's my problem?”, “where's the patient’s part of it?” So, my decision- making has very much changed’ (P19)* |
| Resourcing | *‘That pre-project stuff, that takes a lot of time. And for this one* [physiotherapists completing tertiary-led surveys in a trauma cohort]*, this has been many years of conversations between the physios and the trauma surgeons … The project officer is 0.7 EFT over 16 months - EFT* [effective full-time] *to help with statistical analysis … and we’ve also factored in 0.1 EFT to help us with data collection as well. So that doesn't account for all the steering committee attendance and the in-kind stuff’ (P1)*  *‘They put this new model in, again as a pilot … they looked at our learnings* [from a previous unsuccessful pilot]*, and I think what they took from that was more training, but also dedicated time - which we didn't have, you know, solely dedicated time’ (P2)*  *‘With more money, you have more time that you can spend* [on new scope of practice models]*, mostly on the processes, and there’s got to be a demonstrated pay-off in the end’ (P7)*  *‘That* [an advanced physiotherapy-led osteoarthritis clinic] *also came with funding … That provided us with an opportunity to really get it right, get the communication right. So having project leads, to get it right, get the communication right, get the steering committee around the table, just to make sure that everybody was on the same page. And to make sure it was incremental steps’ (P12)*  *‘Money speaks. It's so much* *easier* [to progress new practice models] *when you've got the money there’ (P13)* |
| Knowledge, skills & experience: clinical | *‘The musculoskeletal roles* [advanced practice physiotherapy roles] *are key examples … When they first kicked off many years ago, you know, ordering imaging was not within scope … but that gradually has come into the scope of practice now, and so it's not really a scope of practice change, but what is it then? It’s just now advanced learning related to image interpretation, so by default for me it’s in scope of practice, it’s just a bigger part of practice today’ (P7)*  *‘Advanced practice has evolved … prescribing for podiatrists, that’s not advanced practice now … because new graduates coming out have an endorsement* [to prescribe set medications]*’ (P11)*  *‘We pitched it at a post-grad level* [advanced musculoskeletal physiotherapy role in the Emergency Department]*, that you’d done your Masters … you’ve got a really strong background in musculoskeletal assessment’ (P11)*  *‘If you look at from screening to assessment, for example, am I okay to screen but not to assess? … Dieticians use a tool called the MST, so it’s about malnutrition and there’s three questions, but they’re all objective and you don’t have to make a clinical decision … Whereas when I look at some of these other tools from other disciplines, then in reality, I am having to make decisions – but is that within my scope?’ (P15)*  *‘The other thing that isn't always clear for us is about when it* [a clinical task currently deemed advanced practice] *… becomes standard scope … People were essentially using the same skills in our Hand Clinic, but then if they were in the Plastics Clinic …they were advanced scope … as a substitution model for the Plastics consultants and registrars’ (P17)* |
| Supporting evidence & resources | *‘I guess the first thing that I would have looked at* [in pursuing an advanced/extended scope model] *… would have been is it already ‘in scope’? … and so just to see whether anyone else was doing it’ (P3)*  *‘The framework of how they set-up pathways for* [musculoskeletal] *advanced practice … we adapted pretty much every one of those documents to still be in the format, but utilise this support material to make the* [vestibular advanced practice] *pathway easier. You know, this is how you do trainee curriculum, this is how you do competency evaluation – and just sub out the content‘ (P7)*  *‘We definitely looked at the data … from ED and patients that were there with falls. What sort of falls? You know, what is their outcome? What was their, sort of, patient journey and did they really need to be in the hospital or not? And hypothesise, you know, hypothesise that we could actually, would have been okay to see them at home, and potentially even at that ambulance level’ (P10)*  *‘There was no project support, no workforce roles or support roles to help you with this* [development of an advanced practice model]*. It was quality improvement methodology on the run by ‘googling’, learning, reflecting … do your homework, do the research, the reading’ (P11)*  *‘There was a very comfortable and focused community of practice* [in musculoskeletal physiotherapy]*. And we knew that there was competition for funding* [across health services]*, but it was OK - this was a “bigger than us” type approach. How do we get the best that we possibly can? … I think we knew that it was going to have, be the foundation and have huge influence if it was successful as we went forward. And if it wasn't successful, well that was sort of an opportunity lost’ (P12)*  *‘I always look at “is there a need?” and “what's the population*?” [in considering an advanced/extended scope of practice models]*. Looking at the epidemiological data and our local area needs analysis, to go, what's it going to look like in the future’ (P19)* |
| Knowledge & skills: change | *‘As you get into more of that advanced scope, you want the seniors* [senior allied health clincians]*, you want them to have not just the clinical skills, you want them to have the other, I suppose, the attributes ... there is something about establishing the scope, then how do you push the envelope? How do you extend that?’ (P10)*  *‘It really came about through, I think, combined interest certainly from the physios, and also from the Emergency Department’ (P12)*  *‘Really, it was the staff first recognising it was an issue … that it was delaying care’ (P15)*  *‘Generally, they're* [allied health clinicians] *pretty onboard, they're actually wanting to put it forward and are looking for those avenues to work at top of scope and they can see where they can value-add. So, it's often out of that frustration of, you know, I had to refer a patient on which means they've got to have another appointment and we could actually do that here’ (P17)*  *‘The rollout of the model was overseen by our Transformation Oversight committee, so that in itself has a particular methodology … it's very regimented in its’ process through to the final outcome’ (P18)* |
| Sustainability | *‘We’ve had advanced prac initiatives here that have fallen over after the fact, so sustainability needs to be considered front and centre’ (P1)*  *‘Thinking about sustainability, what's the volume of patients that require this* [clinical service delivered by advanced/extended scope]*? And if we have one or two clinicians, is there the volume* [of patients] *that they maintain currency* [of skills] *in this space?’ (P1)*  *‘I think that the issue that we were discovering was that, you know, there actually needs to be more training prior to coming into this specific* [advanced practice] *role … and I think that the reason it stopped were the risks were getting higher and higher* [for the allied health clinicians’ skill level]*’ (P2)*  *‘We definitely targeted that* [sustainability] *early on. We made sure that we had a training plan and a training program, a succession plan in place when they started. That's really important … Probably the only one now* [that is less sustainable] *would be prescribing in the Emergency Department because it’s very, very bespoke training that's only available via organised tertiary training, so you have to have a volume of staff … That's probably our most at risk’ (P7)*  *‘If the clinician who drove it and built it* [an advanced/extended scope model] *and made it all happen, and then they leave. You know, there's a sustainability issue there’ (P7)*  *‘What we did from there was go, well, is it within the scope?* [for a specific allied health profession] *… and if it is within the scope, do they need to have further education or not? If it's not in the scope, is it out of scope completely or is it out of scope because we don't have a competency?’ (P10)*  *‘I don't want to see ‘pop-up shops’* [of an advanced/extended scope of practice model] *… We need to actually say, let's do it at that site and let's make sure we have a training and sustainability pathway so it won't fall over, because we shoot ourselves in the foot all the time’ (P19)* |
| Organisational meso-environment | *‘I found it actually quite significantly easier in the hospital* setting [to pursue an extended scope of practice]*, I think because of the safety nets around you. You've got that collegial discussion. Whereas I suppose in community health you’re more isolated’ (P8)*  *‘When you’re thinking about working to the top of your scope, I think a big factor there is probably confidence – and confidence from the organisation as well, that this will be supported and that we're behind the person doing it’ (P9)*  *‘I think as a collective, it would be easier if this was done* [determination of advanced/extended scope of practice] *across the professions rather than, you know, individual little hospitals kind of rolling it out’ (P13)*  *‘‘*[It’s been] *a big change for me from a big metro hospital where there were excellent systems and processes and policies and very clear guidelines around the development of advanced practice roles, to a rural health service that struggles to fill vacant EFT* [effective full time staffing] *let alone put advanced scope roles in’ (P14)*  *‘In trying to influence decisions to happen here* [to implement advanced/extended scope models]*, you know, I will often say “Oh for God’s sake, X* [a comparable health service] *has got this, X do it this way”, and it’s almost shaming. It’s almost like if X can do it, well surely we can do it’ (P20)* |
| Change culture & appetite | *‘Language can affect how we position ourselves in an organisation … the perception of extended* [scope] *is “oh you're working outside of where you should be” – like this is a risky thing to do. Whereas advanced* [practice] *is “it's OK, we’re working at the top of our scope of practice” - it's an easier sell’ (P1)*  *‘I think back to the UK* [in comparison to advanced/extended scope in Australia] *… getting extended scope to take blood, so venepuncture and actually then being allowed to request pathology‘ (P3)*  *‘I do think that we* [allied health] *are sometimes our own worst enemy because we don't jump at things* [scope of practice opportunities]*’ (P9)*  *‘I did work in the UK for a little while, and I don't know, they seem to be possibly less risk adverse* [regarding advanced/extended scope of practice] *over* *there* [in comparison to Australia]*’ (P9)*  *‘We’ve just had a round of* [allied health] *advanced practice grants from the Department of Health in Victoria. And I think, I don’t know if you know anything about them, but it’s pretty concerning how loosely the term advanced practice has now been implemented and the way that these grants were allocated’ (P11)*  *‘The NHS* [National Health Service in the United Kingdom] *were a very great resource* [for allied health advanced/extended scope examples]*, they had been well-versed in scope of practice for many years and just saw it as part of their norm’ (P12)*  *‘Allied health staff have … such an impact on how people manage in hospital settings and how they function as they go home. So, I think we really do need to harness that* [through advanced/extended scope models]*, and I don't think we've worked enough to develop that aspect of value-based care’ (P12)*  *‘I would say* [allied health] *are passive by being disempowered … that’s a really important distinction … they’re seeking some approval and some permission to act and* do [to pursue advanced/extended scopes of practice]*, and they’re looking for direction … not just being resistive’ (P20)* |
| Perceived professional territorialism | *‘The NUMs* [Nurse Unit Managers] *were extremely supportive. The Clinical Service Director was really, really supportive. The Heads of Allied Health were supportive, obviously because they wanted this to work* [an advanced practice model]*. But it was the other disciplines that we got quite a lot of push back from … You know, obviously physio, OT* [occupational therapy]*, social work … it took a long time to get the buy-in* [across allied health professions]*, which is sad actually’ (P2)*  *‘Actually that was quite interesting, and probably at the time controversial, you know, how does physio and OT* [occupational therapy] *share skills? Is it assessment? Is it, you know, is it also progression of care? … Obviously I had some different thoughts and, you know, also conflicting emotions in how I felt about this* [as a physiotherapist]*’ (P10)*  *‘There has been some, I suppose, personality challenges with … how that person* [practising at advanced scope] *is perceived by other disciplines, and possibly some of that is around that unclear scope of practice … that crossed the line into physio territory’ (P14)*  *‘It’s sort of stepping on other professions’ toes so to speak* [implementing an advanced/extended scope of practice] *– you know, what has always been the doctor’s role. And it’s like, well, okay, how do you work around this? How do you … “tippy toe” around this* [to manage impacted stakeholders]*?’ (P15)* |
