# Supplementary figures and images for: The what, the when and the how: A qualitative study of allied health decision‐maker perspectives on factors influencing the development and implementation of advanced and extended scopes of practice in Australia
Source: Int J Health Plann Manage. 2024 Oct 3;40(1):130–55. doi: 10.1002/hpm.3850 (PMC11704828; doi:10.1002/hpm.3850)

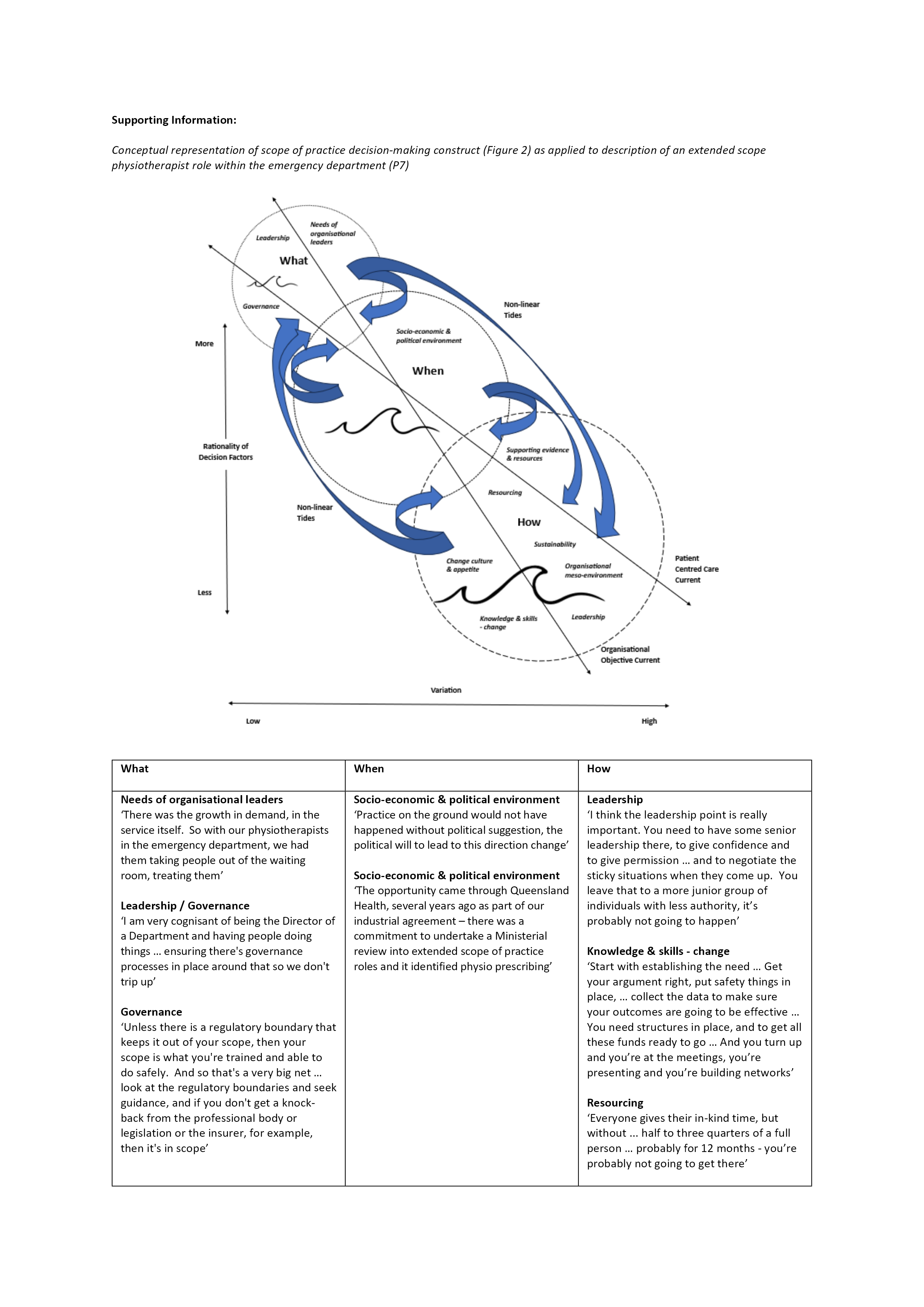

Supplement: Supplementary file 4 — Supporting Information S4 [file HPM-40-130-s001.tiff]

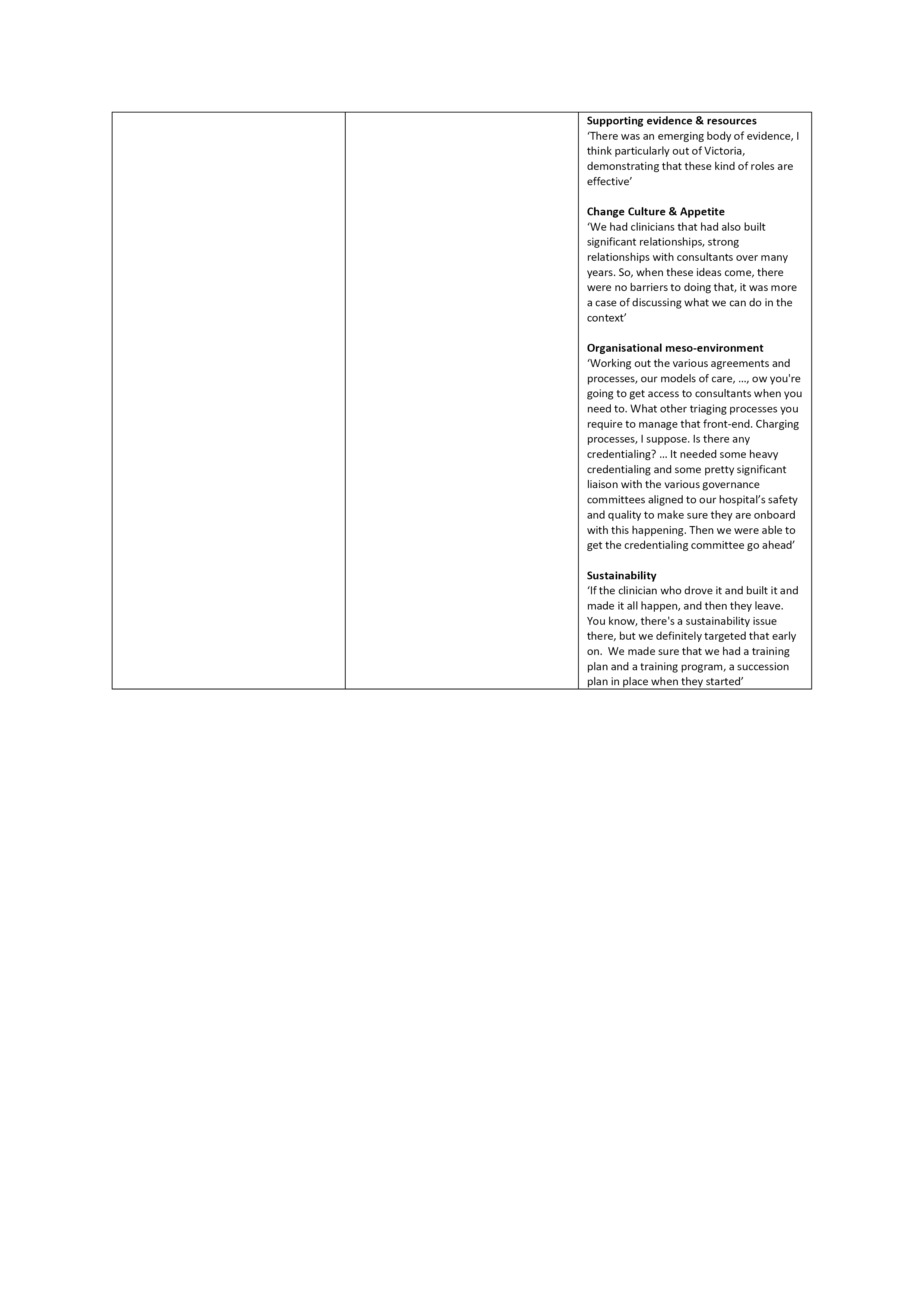

Supplement: Supplementary file 5 — Supporting Information S5 [file HPM-40-130-s002.tiff]
